# The Prevalence of Anemia among Pregnant Women in China: A Systematic Review and Meta-Analysis

**DOI:** 10.3390/nu16121854

**Published:** 2024-06-13

**Authors:** Yalin Zhou, Ying Lyu, Wanyun Ye, Hanxu Shi, Yile Peng, Zhang Wen, Anuradha Narayan, Xiaona Huang, Suying Chang, Yuning Yang, Yajun Xu

**Affiliations:** 1 Department of Nutrition and Food Hygiene, School of Public Health, Peking University, NO.38 Xueyuan Road, Haidian District, Beijing 100083, China; zylyingyang@163.com (Y.Z.); lybjmu@126.com (Y.L.); yewanyun_vera@bjmu.edu.cn (W.Y.); 1610306136@pku.edu.cn (H.S.); 1810306134@pku.edu.cn (Y.P.); 1710306240@pku.edu.cn (Z.W.); 2 PKUHSC-China Feihe Joint Research Institute of Nutrition and Healthy Lifespan Development, NO.38 Xueyuan Road, Haidian District, Beijing 100083, China; 3 Nutrition and Child Development Section, UNICEF Headquarters, 3 United Nations Plz, New York, NY 10017, USA; anarayan@unicef.org; 4 Child Health and Development Section, UNICEF Office for China, NO.12 Sanlitun Road, Chaoyang District, Beijing 100600, China; xhuang@unicef.org (X.H.); schang@unicef.org (S.C.); yyang@unicef.org (Y.Y.); 5 Beijing Key Laboratory of Toxicological Research and Risk Assessment for Food Safety, Peking University, NO.38 Xueyuan Road, Haidian District, Beijing 100083, China

**Keywords:** anemia, iron deficiency, iron deficiency anemia, pregnant women, China

## Abstract

The systematic review and meta-analysis were conducted to ascertain the prevalence of anemia, iron deficiency (ID), and iron deficiency anemia (IDA) among Chinese pregnant women. A total of 722 articles on maternal anemia during pregnancy published between January 2010 and December 2020 were compiled, and a systematic review and meta-analysis were conducted on 57 eligible studies including 1,376,204 pregnant women to ascertain the prevalence of anemia and the prevalence in different subgroups. The results showed that the prevalence of anemia, ID, and IDA among pregnant women in China were 30.7% (95% CI: 26.6%, 34.7%), 45.6% (95% CI: 37.0%, 54.2%), and 17.3% (95% CI: 13.9%, 20.7%), respectively. All prevalence increased with the progression of the pregnancy. There were sizable regional variations in the prevalence of anemia, ID, and IDA. Generally, lower prevalence was observed in the economically more advanced eastern region of the country, while the prevalence of ID was higher in the eastern region than that in the western region. The prevalence of anemia and IDA in rural areas was higher than that in urban areas, but ID prevalence was higher in urban areas. In conclusion, the regional differences and urban–rural disparities in the prevalence of anemia indicate the need for more context-specific interventions to prevent and treat anemia. It was found that dietary factors were one of the major causes of anemia, and iron-containing supplements and nutrition counseling could be effective interventions to reduce the prevalence of anemia, ID, and IDA among Chinese pregnant women.

## 1. Introduction

Anemia is a condition in which the volume of red blood cells in the peripheral circulation is lower than the normal range, obstructing adequate oxygen from being transported to tissues, thus obstructing the normal physiological functions of the body. Anemia significantly affects population health, impairs physical and cognitive growth, and eventually impacts socioeconomic development. It is a serious public health problem globally, especially in low- and middle-income countries [1].

About a quarter of the world’s population suffers from anemia. Children and women, especially pregnant women, are at higher risk [2,3]. Based on different causes, anemia can be classified into three types: nutritional anemia, disease-related anemia, and anemia caused by genetic factors [4]. The deficiency in iron, folate, vitamin B_12_, vitamin A, vitamin C, and other nutrients can cause nutritional anemia, and it is common in low- and middle-income countries [5]. Iron deficiency (ID) is one of the main causes of anemia, accounting for about 50% of all anemic cases [6]. The occurrence of ID and iron deficiency anemia (IDA) can be affected by geographical, cultural, dietary, and economic factors, as well as genetic factors (e.g., single-nucleotide polymorphisms of gene *TMPRSS*6) [7,8]. IDA during pregnancy is associated with adverse health outcomes for both mothers and babies, including premature delivery, infants who are small for gestational age, low birth weight, or even perinatal death [5,9,10]. The common prevention and treatment of ID and IDA during pregnancy include iron-containing supplements, medicines, and nutrition counseling on dietary practices [2,11,12].

In China, some national surveys, routine data reporting systems, and research studies [13,14,15] have assessed the prevalence of anemia among pregnant women in recent years. However, the published data have been inconsistent. Many of the studies were conducted within confined geographical areas, some had small sample sizes or data collected from population-based cross-sectional surveys or management information systems of health facilities, and others did not report data on anemia rates in different trimesters. In this paper, the objective is to conduct a systematic review and meta-analysis using data derived from available studies to establish the prevalence and pattern of ID and IDA among pregnant women across China, in order to inform and facilitate the design and implementation of targeted interventions.

## 2. Methods

**Figure 1:**
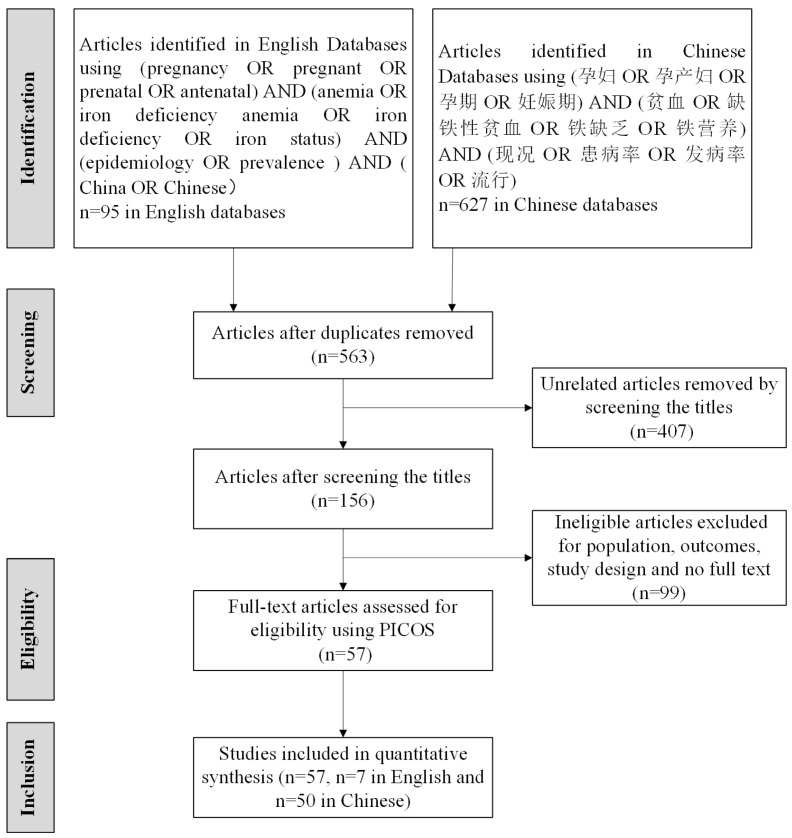
PRISMA flow diagram. 孕妇: pregnant women; 孕产妇: pregnant and maternity women; 孕期 or 妊娠期: pregnancy; 贫血: anemia; 缺铁性贫血: iron deficiency anemia; 铁缺乏: iron deficiency; 铁营养: iron nutrition status; 现况OR患病率 OR 流行: prevalence; 发病率: incidence.

The systematic review and meta-analysis were conducted following the Preferred Reporting Items for Systematic Reviews and Meta-Analyses (PRISMA) guidelines (Figure 1). Two researchers independently searched through English databases (Medline, PubMed, Embase, Web of Science, and Science Direct) and Chinese databases (China National Knowledge Infrastructure, Wanfang, and Chongqing VIP). Relevant studies published between 1 January 2010 and 31 December 2020 were gathered using a combination of Medical Subject Headings (MeSH) and free-text terms including the following: (anemia OR hemoglobin) AND (pregnancy OR maternal OR pregnant OR perinatal OR prenatal) AND (Chinese OR china) AND (prevalence OR epidemiology). The review was not registered, and no protocol was prepared.

### 2.1. Search Strategy and Selection Criteria

Criteria of inclusion and exclusion were applied to the selection of articles; please see Participant, Intervention, Comparison, Outcome Study (PICOs) in Table 1. Two researchers independently reviewed the titles, abstracts, and the full text to exclude studies that did not meet the inclusion criteria. The detailed screening process of articles is shown in Figure 1.

### 2.2. Primary Outcome 

The primary outcomes were the prevalence of anemia, ID, and IDA. The WHO diagnostic criteria of anemia among pregnant women were applied: anemia was defined as a hemoglobin (Hb) level of less than 110 g/L [16], with mild anemia ranging between an Hb level of 100 and 109 g/L, moderate anemia ranging between an Hb level of 70 and 99 g/L, and severe anemia defined as an Hb level of less than 70 g/L [10]. For the diagnosis of ID among pregnant women, the cutoff recommended in the Diagnosis and Treatment of Iron Deficiency and Iron Deficiency Anemia in Pregnancy issued by the Perinatal Branch of the Chinese Medical Association in 2014 was applied: serum ferritin (SF) of less than 20 µg/L. IDA was defined as an Hb level of less than 110 g/L and an SF value of less than 20 µg/L [17].

### 2.3. Quality Assessment and Data Collection

Two researchers independently extracted and reviewed the data from all the selected articles (Table S1), including the authors, year of publication, year of investigation, study location, economic condition of the study location, study design, sampling strategies, sample size, diagnostic criteria of anemia, ID and IDA, prevalence of anemia, ID, and IDA among pregnant women, and whether the Hb level was adjusted according to altitude and smoking status. Any disagreements were resolved by consensus or involving a third researcher. The quality assessment of the studies included in this research was carried out per standard requirements. 

### 2.4. Data Analysis

Firstly, we tested the heterogeneity of prevalence estimates reported in included studies with the Cochran Q test and *I*^2^ index. The heterogeneity existed among studies if Cochran Q showed a *p* < 0.10 and *I*^2^ > 50% [18]. According to the *I*^2^ statistic, heterogeneity was categorized into 3 levels: *I*^2^ < 25% taken as low heterogeneity, 25–75% as moderate heterogeneity, and >75% as high heterogeneity [19]. Then, based on the result of the heterogeneity test, a fixed- or random-effects model was selected for the estimate of the pooled prevalence of anemia, ID, and IDA among pregnant women and 95% confidence intervals (CIs). All statistical analyses were carried out using Stata version 12.0 (StataCorp, College Station, TX, USA) and SPSS version 22.0 (SPSS Inc., Chicago, IL, USA).

## 3. Results

### 3.1. The Characteristics of Eligible Studies

**Figure 2:**
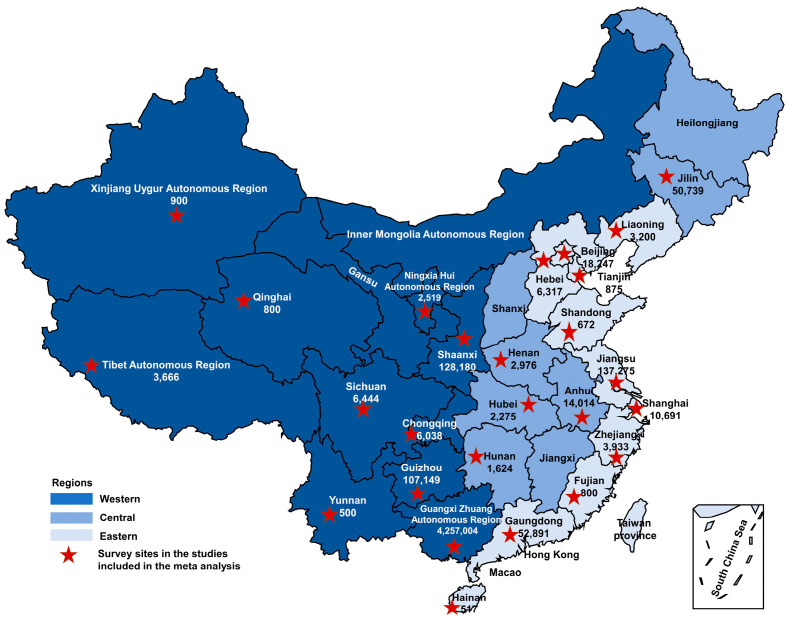
The geographic location of the studies included in the meta-analysis.

A total of 57 studies that reported on the prevalence of anemia, ID, or IDA, with a total sample size of 1,376,204 pregnant women, were included in the meta-analysis. The geographic location of the studies spanned the entire country and covered 23 of China’s 34 provinces, autonomous regions, municipalities, and special administrative regions. According to the National Bureau of Statistics, mainland China can be classified into three regions—eastern, central, and western (Figure 2). Many economic and human development indicators are lower in the western region than in the central and eastern regions [20,21]. In this study, the provinces and municipalities of Hebei [22,23,24], Beijing [13,25,26], Tianjin [27], Shandong [28], Jiangsu [29,30,31,32,33], Shanghai [34], Zhejiang [35,36,37,38], Fujian [39], Guangdong [13,33,40], Hainan [41], and Liaoning [42,43,44] represent the eastern region; the provinces of Henan [45,46], Hubei [47], Hunan [48,49], Anhui [50], and Jilin [42,51,52,53] represent the central region; and the provinces, autonomous regions, and municipalities of Chongqing [54,55,56], Sichuan [13,56,57], Shaanxi [58,59], Yunnan [60], Guizhou [58,61], Guangxi Zhuang Autonomous Region [58,61], Ningxia Hui Autonomous Region [62,63,64,65], Tibet Autonomous Region [66,67], and Xinjiang Uygur Autonomous Region [68] represent the western region. Detailed information on the included studies is described in Tables S2–S4.

### 3.2. Prevalence of Anemia among Pregnant Women in China

#### 3.2.1. Pooled Prevalence

The prevalence of anemia among pregnant women reported by the 36 studies varied widely. The meta-analysis showed that the pooled prevalence of anemia was 30.7% (95% CI: 26.6%, 34.7%). Among anemic cases, roughly half were mild (15.8%; 95% CI: 14.0%, 17.6%), close to 40% were moderate (11.8%; 95% CI: 8.9%, 14.7%), and about 4% were severe (1.1%; 95% CI: 0.8%, 1.5%) (Table 2). Global data show that the prevalence of anemia varies by trimester and geographic location [69]. Therefore, the meta-analysis was conducted to compute the prevalence of anemia among pregnant women in the subgroups as well. However, after the stratification by severity, trimesters, regions, and residence, the *I*^2^ statistic varied from 99.3% to 100%, implying the existence of the heterogeneity among included studies.

#### 3.2.2. Prevalence by Trimester

The physical decrease in Hb concentration occurs during pregnancy because the expansion of plasma volume exceeds the increase in the red blood cell volume. Therefore, the trimester that pregnant women are in is one of the influencing factors of anemia [70]. As shown in Table 2, a higher proportion of women in their third trimester were anemic in comparison with those in the first and second trimesters.

#### 3.2.3. Prevalence by Region and by Residence

The prevalence of anemia among pregnant women varied greatly in different regions (shown in Table 2). The subgroup meta-analysis by region showed an increasing trend in the prevalence of anemia among pregnant women from eastern areas to central and western regions. In addition, the prevalence of anemia among pregnant women residing in rural areas was more than double that of pregnant women in urban areas. However, one exception was found in the data collected in Henan Province where the prevalence of anemia among pregnant women in rural areas was lower than that of pregnant women in urban areas (19.6% in rural areas versus 31.5% in urban areas) [71].

### 3.3. Prevalence of ID among Pregnant Women in China

#### 3.3.1. Pooled Prevalence

A total of 11 studies were included in this meta-analysis on the prevalence of ID among pregnant women in China. The meta-analysis showed that the pooled prevalence of ID was 45.6% (95% CI: 37.0%, 54.2%), suggesting nearly half of the pregnant women in China suffered from ID (Table 3). The heterogeneity existed among studies with *I*^2^ between 98.8% and 99.90%.

#### 3.3.2. Prevalence by Trimester

Based on the data available, it was found that about a quarter of the pregnant women in their first trimester suffered from ID, while slightly over half of the pregnant women in their third trimester suffered from ID.

#### 3.3.3. Prevalence by Region and by Residence

Contrary to the patterns observed in the prevalence of anemia, the prevalence of ID was higher among pregnant women in the eastern region than the western region and higher among pregnant women residing in urban areas than those in rural areas.

### 3.4. Prevalence of IDA among Pregnant Women in China

#### 3.4.1. The Pooled IDA Prevalence

The meta-analysis of the 17 studies included in this research showed that the prevalence of IDA among all pregnant women in China was 17.3% (95% CI: 13.9, 20.7) (Table 4). The heterogeneity existed among studies with *I*^2^ between 98.6% and 99.90%.

#### 3.4.2. Prevalence by Trimester

Similar to the patterns observed in the prevalence of anemia and ID, the subgroup meta-analysis by trimester showed that the prevalence of IDA increased steadily from the first trimester to the third trimester. However, the prevalence of IDA in each trimester was lower than that of anemia and ID in the corresponding trimester.

#### 3.4.3. Prevalence by Region and by Residence

The subgroup meta-analysis by regions was conducted for the pooled prevalence of IDA in the eastern, central, and western regions of China. The prevalence among pregnant women in the central region was higher than both the eastern and western regions. The prevalence of IDA among pregnant women residing in rural areas was found to be nearly twice as high as that in urban areas.

## 4. Discussion

The 57 studies included in this research collectively sampled 1,376,204 pregnant women who lived in 23 of China’s 34 provinces, autonomous regions, municipalities, and special administrative regions. Our analysis found that the prevalence of anemia, ID, and IDA in pregnant women in China varied by region, urban and rural areas, and the trimester. Moreover, this research found that the prevalence of anemia among pregnant women in China was 30.7%, with roughly half of the cases classified as mild. In comparison, the prevalence of ID and IDA were 45.6% and 17.3%, respectively.

### 4.1. Anemia, ID, and IDA Prevalence among Pregnant Women in China

According to the WHO classification [10], the prevalence of anemia among pregnant women in China (30.7%) found during this research would be classified as a “moderate” public health problem (20.0–39.9%). This was higher than the 13.6% stated in the “Report on nutrition and chronic diseases of Chinese residents (2020)” published by the China Nutrition and Health Surveillance (CNHS) [72].

In western China, the prevalence of anemia among pregnant women (38.2%) would be close to being regarded as a “severe” public health problem (>40.0%), despite China’s rapid economic development in recent decades. At the national level, although the prevalence of anemia among pregnant women was lower than the global average (36.5%), it was higher than the prevalence in most BRICS countries that shared a similar developing economic situation with China, namely Brazil (19.1%), Russia (23.4%), India (50.1%), and South Africa (30.8%) [73]. The prevalence of anemia among Western pregnant women (38.2%) was close to that in Southeast Asia (37.5%) but lower than that in South Asia (46.7%) [74]. The GDP per capita in China’s most economically developed cities, such as Beijing, Guangzhou, and Shanghai, is comparable to that of high-income countries, according to classification of the World Bank [75]. Yet, our analysis revealed that the prevalence of anemia in those cities was still much higher (19.3%, 38.8%, and 37.3%, respectively, compared to 17.2% in high-income countries) [13,34]. This indicates that the government of China needs to also allocate funding to prevent maternal anemia in the developed areas of the country.

The high prevalence of ID (45.6%) indicates that nearly half of the pregnant women in China suffer from ID. Significantly, the prevalence in China is much higher than what was found among pregnant women in the second trimester in Switzerland (31.8%) and among pregnant women during the third trimester in America (18.5%) [76,77]. SF is an effective indicator to reflect the status of iron stores. Screening for SF should be routinely performed in pregnant women to trigger intervention to prevent the occurrence of IDA during pregnancy [78]. In addition, our findings also suggest that the iron reserves among women of childbearing age in China could be low before conception, indicating the importance of iron supplementation during the pre-pregnancy period to prevent ID and IDA during pregnancy.

### 4.2. Regional Differences in the Prevalence of Anemia, ID, and IDA among Pregnant Women in China

The significant differences in the prevalence of anemia by region suggest its association with overall socioeconomic status [79], including the quality of medical care services, income levels, dietary diversity and quality, and education level, particularly among pregnant women.

Previous research showed that dietary diversity among residents in rural areas was low, especially in poorer areas where the diet consisted of mainly grains with inadequate intake of animal-based foods and fruits [80,81,82]. One study conducted in the rural areas of northern Shaanxi Province (e.g., western region of China) found that the diet of pregnant women primarily comprised grains and cereals, with an inadequate intake of meat, eggs, milk, fruits, and vegetables [83]. Another study reported that more than 70% of pregnant women had an inadequate intake of fruits and vegetables, and over 90% had a low intake of soybeans, milk, and aquatic products in selected rural poor areas in Anhui Province [84]. In regard to ID, our findings suggest that the prevalence of ID was higher in the economically more developed eastern region and in urban areas, which appeared to be counterintuitive. The following reasons might offer some explanations. Firstly, in terms of screening capacity, only 62.5% of health facilities in China are able to assess SF [85], and fewer facilities in rural areas and the western region had the necessary laboratory equipment, which could lead to the missed reporting of ID cases in these areas. Since ID could lead to IDA and IDA accounted for roughly 50% of anemia globally [1], it is advisable to include SF in the routine testing conducted as part of antenatal and postnatal health care. Secondly, the differences in the varied intake of iron-rich, animal-sourced foods are independent of socioeconomic status. In the eastern region, especially along the coast, the main animal-sourced foods for pregnant women are aquatic foods, instead of red meat, animal offal, and blood, which are richer in heme iron [86]. In comparison, the main animal-sourced foods available in the western region is red meat [87]. For the prevalence of IDA, the regional variation was smaller than that of the prevalence of anemia and ID. Our findings show that the prevalence of IDA in the eastern region of the country is slightly higher than that in the western region. This is consistent, albeit at a smaller magnitude, with the prevalence of ID found in the eastern and western regions.

### 4.3. Other Influencing Factors of Anemia among Pregnant Women in China

Our analysis found that over half (56%) of the cases of anemia in pregnant women were due to ID, which is very close to global estimates [1]. While prioritizing IDA among pregnant women, attention should also be paid to other causes of anemia. The nutritional status of FA among pregnant women has remarkably improved after the national FA supplementation program for preventing neural tube defects took effect in 2009 [88]. Nonetheless, one cross-sectional study conducted in northwestern China showed that about 12.1% of women in late pregnancy were deficient in FA, and 69.6% of the women were deficient in vitamin B12 [89]. According to the CNHS 2015, 1.2% of pregnant women had a vitamin A deficiency in China, a 10.5% marginal deficiency, while only 0.8% of pregnant women in urban areas had a vitamin A deficiency, a 7.5% marginal deficiency [90,91]. The CNHS2010-2012 found that the average intake of vitamin C was 80.1 mg/d among pregnant women, which was lower than the recommended Dietary Reference Intake (100 mg per day) [92]. Thus, a higher percentage of people could have inadequate vitamin C intake in western China, particularly in the northern part of the region (such as Shaanxi Province), where the availability of fresh vegetables and fruits is limited due to prolonged cold weather [59]. Additionally, thalassemia caused by genetic deficiency is prevalent in areas to the south of the Yangtze River, including Guangdong Province, Guangxi Zhuang Autonomous Region, and Yunnan Province. It was reported that 17.4% of pregnant women carry thalassemia genes [93]. A previous study showed that 18.2% of pregnant women in the Guangxi Zhuang Autonomous Region suffered from thalassemia, and routine screening was performed for pregnant women in areas with a high prevalence of thalassemia [94]. Furthermore, clinicians play a crucial role in reducing anemia among pregnant women. Once a pregnant woman is diagnosed with anemia or ID or IDA, doctors should promptly provide appropriate dietary and iron supplementation recommendations to correct anemia and ID or IDA in a timely manner, aiming for a favorable pregnancy outcome. In the literature included in this meta-analysis, doctors in all studies provided appropriate dietary and nutritional supplement recommendations to anemic pregnant women.

### 4.4. Quality of Data and Studies

There remain certain quality issues with the studies included in this research despite the application of the inclusion and exclusion criteria. The Hb values could not be adjusted according to the altitude or the smoking status of the pregnant women in some of studies included, as this information was not collected. Therefore, the prevalence of anemia or IDA could be underestimated. Moreover, there were variations in the methods used to take blood samples and assess Hb values, which may have introduced additional variables in the meta-analysis.

## 5. Recommendations

This meta-analysis and literature review suggest that some program and policy changes could further reduce the prevalence of anemia, ID, and IDA among pregnant women in China. Official standards or guidelines are needed to standardize the lab test protocol and methods for Hb and SF, and SF screening should be a compulsory item in routine antenatal care or basic public health services. Moreover, the must-test items on preventive and therapeutic measures for anemia, ID, and IDA (including iron supplement and nutrition counseling) should be specified in guidelines, appropriate indicators for monitoring should be identified, and the collection, reporting, analysis, and feedback on monitoring data should be required. It is advisable that the official standards or guidelines are issued by the National Health Commission, as this would hold the health services at all levels accountable and have implications for the health insurance scheme. Lastly, prevention and treatment measures, such as multi-micronutrient supplements, should be developed according to the plausible determinants and etiology of anemia among pregnant women residing in different areas in China.

## 6. Conclusions

In conclusion, this study found that the prevalence of anemia among pregnant women in China was 30.7%, with half of the cases categorized as mild (15.8%). During pregnancy, close to half of the women were diagnosed with ID, and close to 20% of them had IDA. In general, the prevalence of anemia, ID, and IDA were higher in the economically less developed western region and in rural areas, with some exceptions for ID. Program and policy changes are recommended to further reduce the prevalence of anemia, ID, and IDA, including the standardization of lab and diagnostic methods, stipulation of indicators and the relevant reporting mechanisms, requirement of SF screening in antenatal care services, and development of preventive and treatment measures tailored to different contexts in China. Additionally, iron-containing supplements and nutrition counseling could be effective interventions to reduce the prevalence of anemia, ID, and IDA among pregnant women in China.

## Figures and Tables

**Table 1:** The inclusion and exclusion criteria for article selection.

PICOS	Inclusions Criteria	Exclusion Criteria
Participants	Pregnant women in China	Non-Chinese pregnant women
Intervention	N/A	N/A
Comparison	N/A	N/A
Outcomes	Prevalence data on maternal anemia during pregnancy	Prevalence data on homeopathy, thalassemia, cancer-related anemia
Study	Original quantitative studies published in peer-reviewed journals	
	Cross-sectional or retrospective studies	Diagnosis criteria for anemia do not follow the WHO guidelines
	Studies that provided the sample size and the number of anemia cases	Sample size less than 400 Studies without full text

N/A: not applicable.

**Table 2:** The prevalence of anemia among pregnant women in China by subgroups.

Subgroup	Number of Studies	Total Sample	Prevalence (%) (95% CI)	Heterogeneity	
				*I^2^*	*p*-Value
By severity					
Mild	15	776,705	15.8 (14, 17.6)	99.90%	0.000
Moderate	14	722,711	11.8 (8.9, 14.7)	100.00%	0.000
Severe	11	766,573	1.1 (0.8, 1.5)	99.70%	0.000
By trimester					
1st TM	19	371,304	17.8 (11.8, 23.7)	99.90%	0.000
2nd TM	21	124,039	31 (23, 38.9)	99.30%	0.000
3rd TM	22	153,928	38.7 (24.8, 52.6)	99.70%	0.000
By region					
East	20	745,152	24 (20, 28.1)	99.90%	0.000
Middle	5	67,729	24.2 (13, 35.5)	99.90%	0.000
West	12	301,300	38.2 (31.1, 45.4)	99.90%	0.000
By residence					
Urban	10	39,442	14.5 (10.4, 18.5)	99.90%	0.000
Rural	14	306,825	34.2 (29.4, 39)	99.90%	0.000
Total	36	1,223,651	30.7 (26.6, 34.7)	100.00%	0.000

**Table 3:** The prevalence of ID among pregnant women in China by subgroups.

Subgroup	Number of Studies	Total Sample	Prevalence (%) (95% CI)	Heterogeneity	
				*I^2^*	*p*-Value
By trimester					
1st trimester	7	2224	23.4 (9.3, 37.4)	99.90%	0.000
2nd trimester	8	7703	38.3 (28.5, 48.2)	99.30%	0.000
3rd trimester	7	7371	51.7 (35.4, 67.9)	99.70%	0.000
By region					
Eastern	4	4297	57.9 (41.8, 74.0)	99.20%	0.000
Central *	1	2275	24.8 (N/A)	N/A	0.000
Western	3	7089	37.2 (28.8, 45.6)	98.80%	0.000
By residence					
Urban	4	18,163	55 (40.6, 69.5)	99.90%	0.000
Rural	2	3288	41.7 (3.6, 79.8)	99.90%	0.000
Total	11	32,917	45.6 (37.0, 54.2)		

* Note: there was only one study on the ID of pregnant women in the central region, which was conducted in Hubei Province. Therefore, no meta-analysis was conducted, and the data were reported here as they were.

**Table 4:** The prevalence of IDA among Chinese pregnant women by subgroups.

Subgroup	Number of Studies	Total Samples	Prevalence (%) (95%CI)	Heterogeneity	
				*I^2^*	*p*-Value
By trimester					
1st trimester	7	5439	5.7 (3.7, 7.6)	99.90%	0.0000
2nd trimester	8	15,821	13.9 (11.1, 16.6)	99.30%	0.0000
3rd trimester	7	20,583	23.9 (18.9, 28.9)	99.70%	0.0000
By region					
Eastern	8	11,283	16.7 (11.3, 22.2)	98.60%	0.0000
Central	3	7007	20.2 (10.8, 29.6)	N/A	N/A
Western	5	13,650	15.5 (7.7, 23.3)	99.50%	0.0000
By residence					
Urban	6	8016	13.3 (6.5, 20.1)	99.90%	0.0000
Rural	4	6161	22.2 (16.9, 27.5)	99.90%	0.0000
Total	17	43,778	17.3 (13.9, 20.7)	99.90%	0.0000

## Data Availability

Datasets are available through the corresponding author upon reasonable request. The data are not publicly available due to privacy reasons.

## Supplementary Materials

The following supporting information can be downloaded at https://www.mdpi.com/article/10.3390/nu16121854/s1; Table S1: Quality assessment questions for the studies included; Table S2: Characteristics of the studies included on the prevalence of anemia among pregnant women in China; Table S3: Characteristics of the studies included on the prevalence of ID among pregnant women in China; Table S4: Characteristics of the studies included on the prevalence of IDA among pregnant women in China.
