# Supplementary material for: The Prevalence of Anemia among Pregnant Women in China: A Systematic Review and Meta-Analysis
Source: Nutrients. 2024 Jun 13;16(12):1854. doi: 10.3390/nu16121854 (PMC11206842; doi:10.3390/nu16121854)
Supplement: Supplementary file 1 [file nutrients-16-01854-s001.zip › nutrients-3046525-supplementary.pdf]

Table S1. Quality assessment questions for the studies included

No.	Author	Q1 Did the author clearly describe the study design?	Q2 Did the author report the sampling method?	Q3 Did the author report the sample size?	Q4 Did the author clearly describe the testing method of hemoglobin (Hb) or serum ferritin?	Q5 Did the author clearly describe the source of the blood samples?	Q6 Did the author adjust the Hb according to smoking status?	Q7 Did the author adjust the Hb according to altitude if applicable?	Q8 Did the author report the demographic characteristics of the pregnant women?	Q9 Did the author report the child-bearing history of the pregnant women?	Q10 Did the author report the prevalence of anemia/ID/IDA in different trimesters, respectively?
1	Yang	Y	Y	Y	N	N	N	N/A	Y	Y	N
2	Li Sun	Y	Y	Y	N	N	N	N/A	N	N	N
3	Mingming Xie	Y	Y	Y	N	N	N	N/A	Y	Y	N
4	Yi Lan	Y	Y	Y	N	N	N	N/A	Y	N	N
5	Huanhuan Wang	Y	Y	Y	N	N	N	N	Y	Y	N
6	Pei Feng	Y	Y	Y	Y	Y	N	N/A	Y	Y	Y
7	Hua Gao	Y	Y	Y	Y	N	N	N/A	Y	N	N
8	Shan Jiang	Y	Y	Y	Y	Y	N	Y	Y	Y	Y
9	Tan	Y	Y	Y	N	N	N	N/A	Y	Y	Y
10	Shufen Ning	Y	Y	Y	Y	Y	excluded women who smoked	N/A	Y	N	N
11	Miao Li	Y	Y	Y	N	N	N	N/A	Y	N	Y
12	Cuiyan Chen	Y	Y	Y	N	N	N	N/A	Y	Y	N
13	Liu Yang	Y	Y	Y	N	N	N	N/A	Y	N	Y
14	Yanhong Xiao	Y	Y	Y	Y	Y	N	N/A	Y	Y	Y
15	Guixiang Huang	Y	Y	Y	N	N	N	N/A	Y	Y	Y
16	Li	Y	Y	Y	N	N	N	N/A	Y	Y	Y
17	Jingli Fu	Y	Y	Y	N	N	N	N/A	Y	N	Y
18	Xiaohua Luo	Y	Y	Y	Y	Y	N	N/A	Y	Y	N
19	Minjie Qi	Y	Y	Y	Y	Y	N	N/A	Y	Y	Y
20	Linli Qian	Y	Y	Y	Y	N	N	N/A	Y	Y	Y
21	Xinyu Liu	Y	Y	Y	N	N	N	N/A	Y	Y	N
22	Wenjun Li	Y	Y	Y	Y	Y	N	N/A	Y	N	Y
23	Jingge Wang	Y	Y	Y	Y	Y	N	N/A	Y	N	N
24	Fen Li	Y	Y	Y	N	N	N	N/A	Y	Y	N
25	Xianxian Zhou	Y	Y	Y	N	N	N	N	Y	Y	N
26	Ying He	Y	Y	Y	N	N	N	N	Y	Y	Y
27	Hongyan Wang	Y	Y	Y	N	Y	N	N/A	N	N	Y
28	Xianfeng Li	Y	Y	Y	Y	Y	N	N/A	Y	N	Y
29	Lin Tang	Y	Y	Y	Y	Y	N	N/A	Y	N	Y
30	Hu	Y	Y	Y	N	N	N	N/A	Y	Y	Y
31	Wenjuan Kong	Y	Y	Y	N	N	N	N/A	Y	N	N
32	Yingying Shao	Y	Y	Y	N	N	N	N/A	Y	N	Y
33	Ping Shi	Y	Y	Y	Y	N	N	N/A	N	N	Y
34	Xia Fan	Y	Y	Y	N	Y	N	N/A	Y	Y	Y
35	Hui Yang	Y	Y	Y	N	N	N	N	Y	Y	N
36	Yijun Kang	Y	Y	Y	Y	Y	N	Y	Y	Y	Y
37	Jun Yu	Y	Y	Y	Y	Y	N	N/A	Y	N	N
38	Lingjue Yu	Y	Y	Y	Y	Y	N	N/A	N	N	N
39	Shuang Lu	Y	Y	Y	Y	Y	N	N/A	Y	Y	Y
40	Wu	Y	Y	Y	Y	Y	N	Y	Y	Y	N

41	Nurbiyah Deshati	Y	Y	Y	N	Y	N/A	N/A	Y	Y	Y
42	Haihong Liu	Y	Y	Y	Y	Y	N/A	N/A	Y	Y	Y
43	Hongmei Yang	Y	Y	Y	Y	Y	N/A	N/A	Y	Y	Y
44	Chenyin Luo	Y	Y	Y	Y	Y	N/A	N/A	Y	N	Y
45	Collaboration group of iron deficiency epidemiological survey among Chinese children, pregnant and reproductive-aged women	Y	Y	Y	Y	Y	N/A	N/A	Y	Y	Y
46	Mingming Li	Y	Y	Y	Y	Y	N/A	N/A	Y	Y	Y
47	Guolin He	Y	Y	Y	N	N	N/A	N/A	Y	Y	Y
48	Liu Kuang	Y	Y	Y	Y	N	N/A	N/A	N	N	N
49	Ling Huang	Y	Y	Y	Y	Y	N/A	N/A	N	N	Y
50	Ma	Y	Y	Y	N	Y	N/A	N/A	Y	N	N
51	Ming Lan	Y	Y	Y	Y	N	N/A	N/A	Y	Y	N
52	Yuan	Y	Y	Y	Y	N	N/A	N/A	Y	Y	N
53	Tiaobing Wang	Y	Y	Y	N	N	N	N/A	Y	Y	Y
54	Can Ye	Y	Y	Y	Y	N	N	N/A	Y	Y	Y
55	Jinli Gao	Y	Y	Y	Y	Y	N	N	Y	Y	Y
56	Jia Liu	Y	Y	Y	N	N	N	N/A	Y	Y	Y
57	Qinghua Ma	Y	Y	Y	N	N	N	N	Y	N	N

Q: question, N/A: not applicable, N: no reporting, Y: yes

Table S2. Characteristics of the studies included on the prevalence of anemia among pregnant women in China

No.	Author	Study period (year)	Study design	Study location	Survey subject	Sample size	Sampling method	Lab assay method	Source of blood sample	Average (%)	1st Trimester (%)	2nd Trimester (%)	3rd Trimester (%)	*Adjust Hb according to altitude level	*Adjust Hb according to smoking status
1	Yang	2012-2015	Retrospective study	Anhui, Central China	Pregnant woman	14,014	Convenience sampling	No	No	8.57				N/A	No
2	Li Sun	2017	Retrospective study	Jiangsu, Eastern China	Pregnant and puerperal woman	134,252	Annual report data	No	No	10.01				N/A	No
3	Mingming Xie	2012-2019	Retrospective study	Chongqing, Western China	Pregnant and puerperal woman	5,038	Convenience sampling	No	No	12.72				N/A	No
4	Yi Lan	2012-2013	Retrospective study	Chongqing, Western China	Pregnant women	1,000	Convenience sampling	No	No	13.20				N/A	No
5	Huanhuan Wang	2012-2014	Cross-sectional study	Three western provinces - Guangxi, Guizhou, Shaanxi ,Western China	Pregnant and puerperal woman	660,651	Surveillance data for high- risk pregnant women in the hospital system	No	No	13.28				N/A	No
6	Pei Feng	2017-2019	Cross-sectional study	Jiangsu, Eastern China	Pregnant women	1,026	Convenience sampling	Colorimetric assay	Venous blood	15.30	5.50	22.04	18.72	N/A	No
7	Hua Gao	2017	Cross-sectional study	Jiangsu, Eastern China	Pregnant women	1,997	Surveillance data of anemia rate	Cyanide methemoglobin	No	16.52				N/A	No
8	Shan Jiang	2010-2012	Cross-sectional study	Nationwide	Pregnant women	3,501	Multistage stratified cluster random sampling	Cyanide methemoglobin	Venous blood	17.20	9.90	17.40	21.50	Yes	No
9	Tan	2020	Cross-sectional study	Six sub-regions of China	Pregnant women	12,403	Multistage stratified cluster random sampling	No	No	19.84	4.91	16.55	23.21		No
10	Shufen Ning	2014-2015	Cross-sectional study	Hebei, Eastern China	Pregnant women	1,856	Convenience sampling	Full-automatic hematology analysis	Venous blood	19.94				N/A	Yes
11	Miao Li	2018	Retrospective study	Hebei, Eastern China	Pregnant women	1,000	Convenience sampling	No	No	20.20	10.38	17.52	30.63	N/A	No
12	Cuiyan Chen	2010-2015	Cross-sectional study	Guangdong, Eastern China	Pregnant women and puerperal women	51,327	Convenience sampling	No	No	20.53				N/A	No
13	Liu Yang	2009	Retrospective study	Liaoning, Eastern China	Pregnant women	3,200	Convenience sampling	No	No	21.63	6.31	20.71	35.65	N/A	No
14	Yanhong Xiao	2016-2018	Retrospective study	Tianjin, Eastern China	Pregnant women	875	Convenience sampling	Sodium lauryl sulfonate	Venous blood	21.94	10.26	21.39	39.44	N/A	No
15	Guixiang Huang	2010-2011	Retrospective study	Hainan, Eastern China	Pregnant women	517	Convenience sampling	No	No	23.02	5	25.79	20.11	N/A	No
16	Li	2013-2015	Retrospective study	Beijing, Eastern China Guangzhou, Eastern China	Pregnant women	43,403	Convenience sampling	No	No	23.50	2.70	14.70	16.6	N/A	No

				Chengdu, Western China											
17	Jingli Fu	2019	Cross-sectional study	Fujian, Eastern China	Pregnant women	800	Convenience sampling	No	No	24.13				N/A	No
18	Xiaohua Luo	2017	Cross-sectional study	Henan, Central China	Pregnant women	438	Convenience sampling	Electrical resistance hematology analysis	Venous blood	24.43				N/A	No
19	Minjie Qi	2015	Cross-sectional study	Henan, Central China	Pregnant women	450	Convenience sampling	Cyanide methemoglobin	Venous blood	24.71	13.18	25.15	35.04	N/A	No
20	Linli Qian	2018-2019	Cross-sectional study	Zhejiang, Eastern China	Pregnant women	550	Convenience sampling	Full-automatic hematology Analy	No	26.70		17.00	26.72	N/A	No
21	Xinyu Liu	2017	Cross-sectional study	Jilin, Central China	Pregnant women and puerperal women	50,739	Surveillance data for high risk-pregnant women system in Jilin province	No	No	26.78				N/A	No
22	Wenjun Li	2010	Cross-sectional study	Hebei, Eastern China	Pregnant women	1,558	Convenience sampling	Full-automatic hematology analysis	Venous blood	27.09	1.82	30.04	35.16	N/A	No
23	Jingge Wang	2013-2014	Retrospective study	Ningxia Hui Autonomous Region, Western China	Pregnant women	1,823	Convenience ampling	Full-automatic hematology analysis	Venous blood	31.43				No	No
24	Fen Li	2015-2018	Retrospective study	Hebei, Eastern China	Pregnant women	1,903	Convenience Sampling	No	No	33.74				N/A	No
25	Xianxian Zhou	2017-2018	Cross-sectional study	Tibet, Western China	Pregnant women and puerperal women	2,136	Convenience sampling	No	No	33.80				No	No
26	Ying He	2013-2015	Cross-sectional study	Ningxia Hui Autonomous Region, Western China	Pregnant women	696	Convenience sampling	No	No	34.77	10.26	28.33	44.86	No	No
27	Hongyan Wang	2013-2015	Cross-sectional study	Shandong, Eastern China	Pregnant women	672	Convenience sampling	No	Venous blood	36.01	24.48	29.63	51.90	N/A	No
28	Xianfeng Li	2009-2011	Cross-sectional study	Henan, Central China	Pregnant women	2,088	Convenience sampling	Full-automatic hematology analysis	Capillary blood	36.78	17.81	35.77	54.20	N/A	No
29	Lin Tang	2016	Retrospective study	Shanghai, Eastern China	Pregnant women	10,691	Convenience sampling	Full-automatic hematology analysis	Venous blood	37.30	4.50	28.70	23.70	N/A	No
30	Hu	2020	Retrospective study	Eastern China: Liaoning, Fujian; Central China: Hebei, Hunan; Western China: Sichuan, Yunnan	Pregnant women	206,753	Data from the Maternal and Newborn Health Monitoring System	No	No	41.98	7.66	45.07	76.03	Yes	No
31	Wenjuan Kong	2016-2019	Retrospective study	Zhejiang, Eastern China	Pregnant women and puerperal	2,000	Convenience sampling	No	No	42.05				N/A	No

					women										
32	Yingying Shao	2017-2018	Retrospective study	Zhejiang, Eastern China	Pregnant women	1,383	Convenience sampling	No	No	48.95	7.74	25.45	15.76	N/A	No
33	Ping Shi	2015-2016	Cross-sectional study	Xinjiang Uygur Autonomous Region , Western China	Pregnant women	900	Convenience sampling	Full-automatic hematology analysis	No	61.07	54.58	53.55	74.11	N/A	No
34	Xia Fan	2011	Cross-sectional study	Shaanxi, Western China	Pregnant women	457	Convenience sampling	No	Venous blood	71.12	58.33	72.34	73.75	No	No
35	Hui Yang	2011-2012	Retrospective study	Yunnan, Western China	Pregnant women and puerperal women	500	Convenience sampling	No	No	78.80				No	No
36	Yijun Kang	2008-2011	Cross-sectional study	Tibet Autonomous Region, Western China	Pregnant women	1,530	Convenience sampling	Automatic hemoglobin meter	Capillary blood	85.60	79.50	86.10	87.30	Yes	No
37	Jun Yu	2009	Cross-sectional study	Liaoning, Eastern China	Pregnant women	1,266	Cluster random sampling	Full-automatic hematology analysis	Venous blood			35.22	53.52	N/A	No
38	Lingjue Yu	2016	Cross-sectional study	Jiangsu, Eastern China	Pregnant women	1,225	Convenience sampling	Full-automatic hematology analysis	Venous blood			17.88	27.35	N/A	No
39	Shuang Lu	2014-2016	Retrospective study	Beijing, Eastern China	Pregnant women	5,240	Convenience sampling	Full-automatic hematology analysis	Venous blood				21.60	N/A	No
40	Wu	2018	Cross-sectional study	Southwest region	Pregnant women and women of childbearing age	144,346	Data from the National Free Preconception Health Examination Project and electronic medical records of local hospitals	Cyanide methemoglobin	Venous blood	41.98	7.66	45.07	76.03	Yes	No

*Refers to WHO's recommendation that the Hb level at an altitude of >1000m should be adjusted according to the altitude. The study excluded the effects of smoking.

There are six sub-regions in China: North, Northeast, East, Central, Southwest and Northwest.

TM: trimester, N/A: not applicable, No: no reporting

Table S3. Characteristics of the studies included on the prevalence of ID among pregnant women in China

No.	Author	Study period (year)	Study design	Study location	Study subjective	Sample size	Sampling method	Lab assay method	Blood sample collection	Diagnostic criteria	Average ID rate	1st Trimester	2nd Trimester	3rd Trimester
1	Nurbiyah Deshati	2018	Cross-sectional study	Wuhan, Central China	Pregnant women	2,275	Convenience sampling	No	Venous blood	20	24.79	19.01	25.63	31.67
2	Yanhong Xiao	2016-2018	Retrospective study	Tianjin, Eastern China	Pregnant women	875	Convenience sampling	Chemiluminescent immunoassay	Venous blood	13	24.80			
3	Xiaohua Luo	2017	Cross-sectional study	Henan, Central China	Pregnant women	438	Convenience sampling	Electrochemiluminescence	Venous blood	10	27.60			
4	Haihong Liu	2017	Cross-sectional study	Shaanxi, Eastern China	Pregnant women	4,548	Multistage stratified cluster random sampling	Chemiluminescent immunoassay	Venous blood	20	28.58	18.53	25.52	31.45
5	Hongmei Yang	2016	Cross-sectional study	Sichuan and Chongqing, Western China	Pregnant women	2,077	Convenience sampling	Chemiluminescent immunoassay	Venous blood	20	28.79	6.13	17.13	39.59
6	Chenyin Luo	2017-2018	Cross-sectional study	Guangdong, Eastern China	Pregnant women	1,280	Convenience sampling	Chemiluminescent immunoassay	Venous blood	11-306.8	41.70	30.60	46.70	58.20
7	Collaboration group of iron deficiency epidemiological survey among Chinese children, pregnant and reproductive-aged women	2000	Cross-sectional study	Nationwide	Pregnant women	3,591	Stratified random sampling	Radioimmunoassay	Serum	20	42.60	39.90	38.80	51.60
8	Mingming Li	2018-2019	Retrospective study	Liaoning, Eastern China	Pregnant women	1,562	Convenience sampling	Chemiluminescent immunoassay	Venous blood	20	44.30	19.08	36.98	52.28
9	Pei Feng	2017-2019	Cross-sectional study	Jiangsu, Eastern China	Pregnant women	1,026	Stratified random sampling	Chemiluminescent immunoassay	Venous blood	20	46.10			
10	Shufen Ning	2013-2014	Cross-sectional study	Hebei, Eastern China	Pregnant women	1,856	Convenience sampling	No	No	12	46.44	7.79	33.33	80.00
11	Guolin He	2016	Cross-sectional study	Six sub-regions	Pregnant women and puerperal women	12,403	Multistage stratified cluster random sampling	No	No	20	48.16			
12	Liu Kuang	2018	Cross-sectional study	Sichuan, Western China	Pregnant women and puerperal women	464	Convenience sampling	Chemiluminescent immunoassay	No	20	51.90			

13	Jingli Fu	2019	Cross-sectional study	Fujian, Eastern China	Pregnant women	800	Convenience sampling	No	No	20	64.00	27.27	61.22	69.72
14	Ling Huang	2016	Cross-sectional study	Liaoning, Eastern China	Pregnant women	909	Convenience sampling	Radioimmunoassay	Venous blood	20	77.23	34.62	54.55	85.40
15	Ma	1999-2001	Cross-sectional study	Gansu, Western China Guangxi Zhuang Autonomous Region , Western China Shandong, Eastern China Fujian, Eastern China	Pregnant women	734	Cluster random sampling	No	Venous blood	12				44.70
16	Ming Lan	2013-2014	Retrospective study	Jiangsu, Eastern China	Pregnant women	3,262	Convenience sampling	Chemiluminescent immunoassay	No	20			47.98	
17	Yuan	2016-2017	Retrospective study	Guangdong, Eastern China Jiangsu, Eastern China	Pregnant women	11,569	Convenience sampling	Chemiluminescent immunoassay	No	12				54.27
18	Linli Qian	2018-2019	Cross-sectional study	Jiangsu, Eastern China	Pregnant women	550	Convenience sampling	Chemiluminescent immunoassay	No	10			47.60	

There are six sub-regions in China: North, Northeast, East, Central, Southwest and Northwest. No: no reporting

Table S4. Characteristics of the studies included on the prevalence of IDA among pregnant women in China

No.	Author	Study period	Study design	Study location	Study subject	Sample size	Sampling method	Lab assay method-Hb	Lab assay method-SF	Blood sample collection	Average	1st Trimester	2nd Trimester	3rd Trimester
1	Tiaobing Wang	2013	Retrospective study	Beijing, Eastern China	Pregnant woma	588	Convenience sampling	No	No	No	3.1	2.4	3.3	3.0
2	Hongmei Yang	2016	Cross-sectional study	Sichuan and Chongqing, Western China	Pregnant woma	2,077	Convenience sampling	Full-automatic hematology analysis	Chemiluminescent immunoassay	Venous blood	5.01	0.5	2.8	7.1
3	Pei Feng	2017-2019	Cross-sectional study	Jiangsu, Eastern China	Pregnant women	1,026	Stratified random sampling	No	No	No	10.0	1.8	11.8	15.0
4	Can Ye	2019-2020	Cross-sectional study	Hunan, Central China	Pregnant women	1,624	Convenience sampling	Full-automatic hematology analysis	Chemiluminescent immunoassay	No	10.8	3.7	6.2	31.1
5	Guolin He	2016	Cross-sectional study	Six sub-regions	Pregnant woma	12,403	Multistage stratified sampling	Colorimetric assay	Chemiluminescent immunoassay	Venous blood	13.9	2.0	8.4	17.8
6	Liu Kuang	2018	Cross-sectional study	Sichuan, Western China	Pregnant women and puerperal women	464	Convenience sampling	Full-automatic hematology analysis	Chemiluminescent immunoassay	No	15.1			
7	Ling Huang	2016	Cross-sectional study	Liaoning, Eastern China	Pregnant woma	909	Convenience sampling	Full-automatic hematology analysis	Radioimmunoassay	Venous blood	17.5	7.7	14.1	18.8
8	Chenyin Luo	2017-2018	Cross-sectional study	Guangdong, Eastern China	Pregnant woma	1,280	Convenience sampling	Hematology analysis	Chemiluminescent immunoassay	Venous blood	17.7	11.6	20.7	26.0
9	Collaboration group of iron deficiency epidemiological survey among Chinese children, pregnant and reproductive-aged women	2000	Cross-sectional study	Nationwide	Pregnant woma	3,591	Stratified sampling	cyanide methemoglobin	radioimmunoassay	capillary blood	19.1	9.6	19.8	33.8
10	Shufen Ning	2013-2014	Cross-sectional study	Hebei, Central China	Pregnant woma	1,856	Convenience sampling	No	No	No	19.9	1.3	9.7	38.5
11	Jinli Gao	2013-2015	Cross-sectional study	Ningxia Hui Autonomous Region, Western China	Pregnant woma	2,405	Convenience sampling	Hematology Analysis	No	Capillary blood	21.8	18.8	14.7	24.5
12	Jingli Fu	2019	Cross-sectional study	Fujian, Eastern China	Pregnant women	800	Convenience sampling	No	No	No	22.3	11.4	20.4	24.4
13	Haihong Liu	2017	Cross-sectional study	Shaanxi, Western China	Pregnant woma	4,548	Stratified cluster random sampling	Full-automatic hematology Analysis	Chemiluminescent immunoassay	Venous blood	24.7	5.2	14.5	32.8
14	Nurbiyah Deshat	2018	Cross-sectional study	Hebei, Central China	Pregnant woma	2,275	Convenience sampling	No	No	Venous blood	24.8	4.9	14.6	32.9
15	Jia Liu	2012	Cross-sectional study	Jilin, Central China	Pregnant woma	3,108	cluster random sampling	No	No	No	24.9	8.9	22. 68	30. 99
16	Mingming Li	2018-2019	Retrospective study	Liaoning, Eastern China	Pregnant women	1,562	Convenience sampling	Full-automatic hematology Analysis	Chemiluminescent immunoassay	Venous blood	26.8	3.8	19.3	34.5

17	Ming Lan	2013-2014	Retrospective study	Jiangsu, Eastern China	Pregnant woma	3,262	Convenience sampling	Full-automatic hematology Analysis	Chemiluminescent immunoassay	Venous blood			10.6	16.9
18	Linli Qian	2018-2019	Cross-sectional study	Jiangsu, Eastern China	Pregnant women	550	Convenience sampling	Full-automatic hematology Analysis	Chemiluminescent immunoassay	No			9.3	15.8
19	Qinghua Ma	2016-2017	Case-control study	Qinghai, Western China	Pregnant women	800	Convenience sampling	No	No	No				22.5

There are six sub-regions in China: North, Northeast, East, Central, Southwest and Northwest.

No: no reporting
